# Two Cases of *Curvularia geniculata* Keratitis Successfully Treated with Natamycin-Based Therapy

**DOI:** 10.1007/s11046-025-00997-9

**Published:** 2025-09-13

**Authors:** Atsuhiko Fukuto, Fumiya Miyako, Toshinori Hara, Rie Nagaoka, Takashi Yaguchi, Hirokazu Sakaguchi, Taiichiro Chikama

**Affiliations:** 1 Department of Ophthalmology and Visual Sciences, Graduate School of Biomedical and Health Sciences, Hiroshima University, Hiroshima, 734-8551 Japan; 2 Section of Clinical Laboratory, Division of Clinical Support, Hiroshima University Hospital, Hiroshima, 734-8551 Japan; 3 Division of Laboratory Medicine, Hiroshima University Hospital, Hiroshima, 734-8551 Japan; 4 Medical Mycology Research Center, Chiba University, Chiba, 260-8673 Japan

**Keywords:** *Curvularia geniculata*, Fungal keratitis, Natamycin, Voriconazole

## Abstract

This report describes two cases of *Curvularia geniculata* keratitis, a rare form of fungal keratitis successfully managed with natamycin-based therapy. Both patients presented with characteristic feathery corneal infiltrates following ocular trauma. In vivo confocal microscopy and direct microscopy revealed septate filamentous fungi, and the isolates were definitively identified as *C. geniculata* through sequence analysis of the translation elongation factor 1-alpha (*tef1-α*) gene. Antifungal susceptibility testing showed sensitivity to natamycin at 2 μg/mL for both isolates, with variable sensitivity to other antifungal agents. Patient 1 was treated with a combination of topical natamycin and voriconazole, while Patient 2 received natamycin monotherapy. Both patients achieved complete healing and excellent visual outcomes. These cases underscore the importance of accurate molecular identification for species differentiation within the *Curvularia* genus and demonstrate the efficacy of natamycin-based therapy for *C. geniculata* keratitis. The choice between monotherapy and combination therapy may be guided by clinical severity and antifungal susceptibility testing. This report contributes to the understanding of the clinical features, diagnosis, and management of this rare condition and highlights the potential value of susceptibility testing in guiding treatment decisions.

## Introduction

Fungal keratitis remains a significant cause of visual impairment worldwide, with dematiaceous fungi—including *Curvularia* species—emerging as important pathogens [1]. These pigmented filamentous fungi are widely found in soil and vegetation, and keratitis often follows plant-related trauma, especially in tropical and subtropical regions [2]. Although several *Curvularia* species have been implicated in ocular infections, keratitis caused by *Curvularia geniculata* is relatively rare, and current knowledge of its clinical features and treatment remains limited.

We report two cases of *C. geniculata* keratitis that were successfully treated with medical management. By presenting these cases, we aim to contribute to a better understanding of the clinical features, diagnosis, and management of this relatively uncommon form of fungal keratitis.

## Case Reports

### Patient 1

**Fig. 1:**
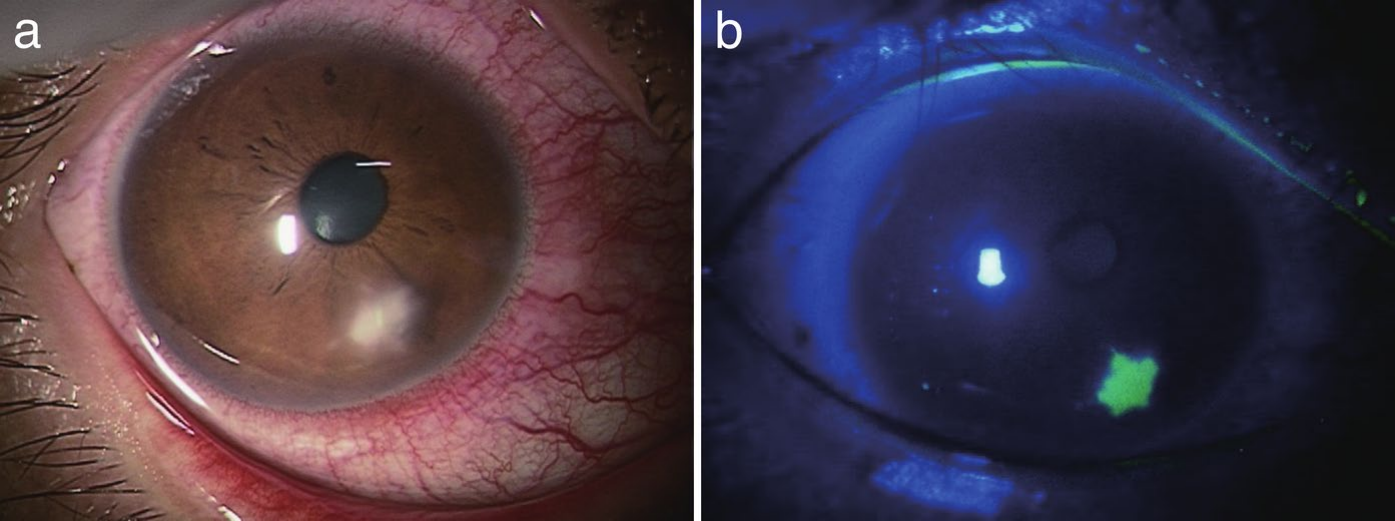
**a** Anterior segment photograph of Patient 1 at the initial examination, showing a 2-mm corneal infiltrate with characteristic feathery borders at the 4-o’clock position. **b** Fluorescein staining demonstrating an epithelial defect corresponding to the area of corneal infiltration

**Fig. 2:**
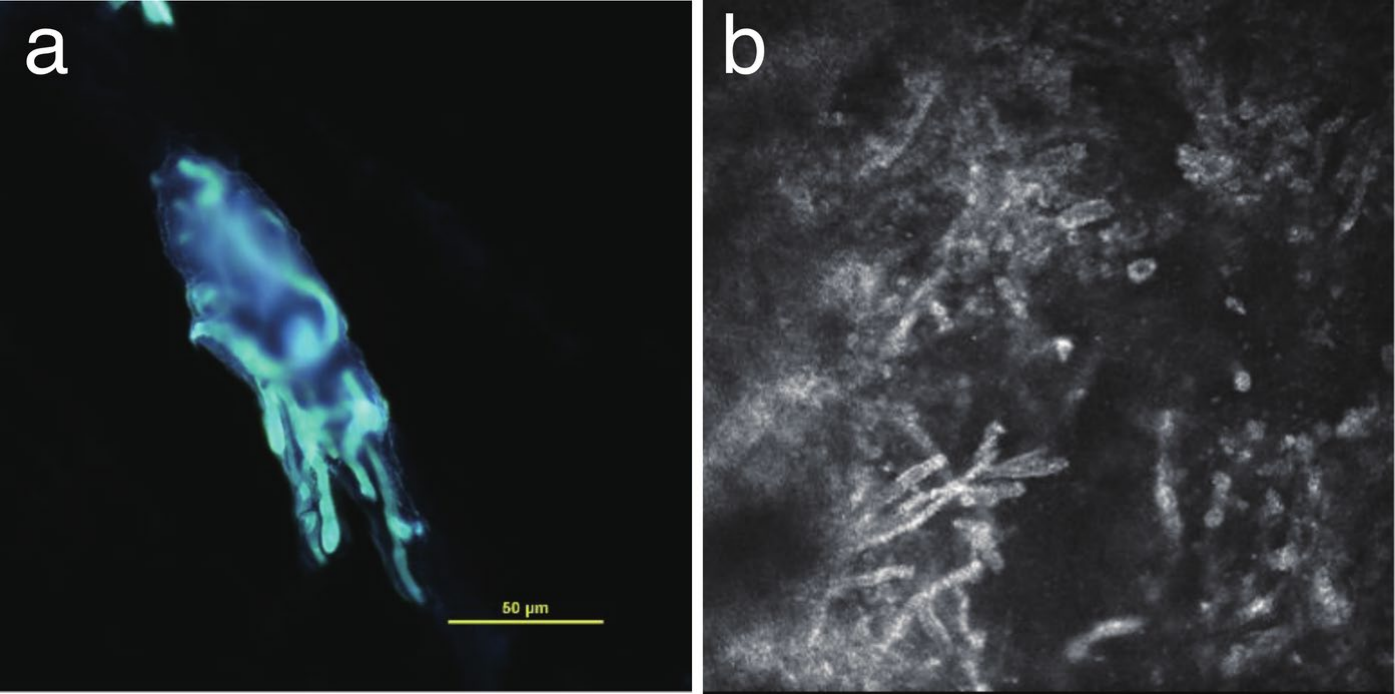
**a** Fungiflora Y staining of a corneal scraping from Patient 1, showing club-shaped, thick, septate filamentous fungi. **b** In vivo confocal microscopy of Patient 1 demonstrating numerous filamentous fungi with morphology similar to that observed on Fungiflora Y staining

A 29-year-old man who worked at an incineration facility crushing garbage presented with hyperemia and a foreign body sensation in his right eye. After visiting a local ophthalmologist the following day, he was referred to the Department of Ophthalmology at Hiroshima University Hospital 5 days later because of lack of improvement. At the initial examination, his corrected visual acuity in the right eye was 20/20. Conjunctival injection was noted, and slit-lamp examination revealed a 2-mm diameter corneal ulcer with characteristic feathery infiltrate at the 4-o'clock position of the right cornea, extending approximately 50% into the stromal depth without corneal thinning or perforation. The lesion was located 2 mm from the limbus in the peripheral cornea (Fig. 1a). The initial corneal scraping did not reveal any organisms. Treatment was started with topical antibiotics: 1.5% levofloxacin six times daily, cefmenoxime six times daily, and ofloxacin ointment three times daily. Three days later, the infiltration had expanded. Subsequent epithelial curettage with fluorescent and lactophenol cotton blue staining revealed club-shaped, thick nodular filamentous fungi (Fig. 2a), and in vivo confocal microscopy confirmed the presence of similarly shaped filamentous fungi (Fig. 2b). On the basis of these findings, fungal keratitis was diagnosed. The treatment regimen was switched to 5% natamycin twice daily and 1% voriconazole four times daily, with 1.5% levofloxacin reduced to four times daily. The other antibiotics (cefmenoxime and ofloxacin ointment) were discontinued. After 10 days of antifungal therapy, the lesion had scarred, and all medications were discontinued.

### Patient 2

**Fig. 3:**
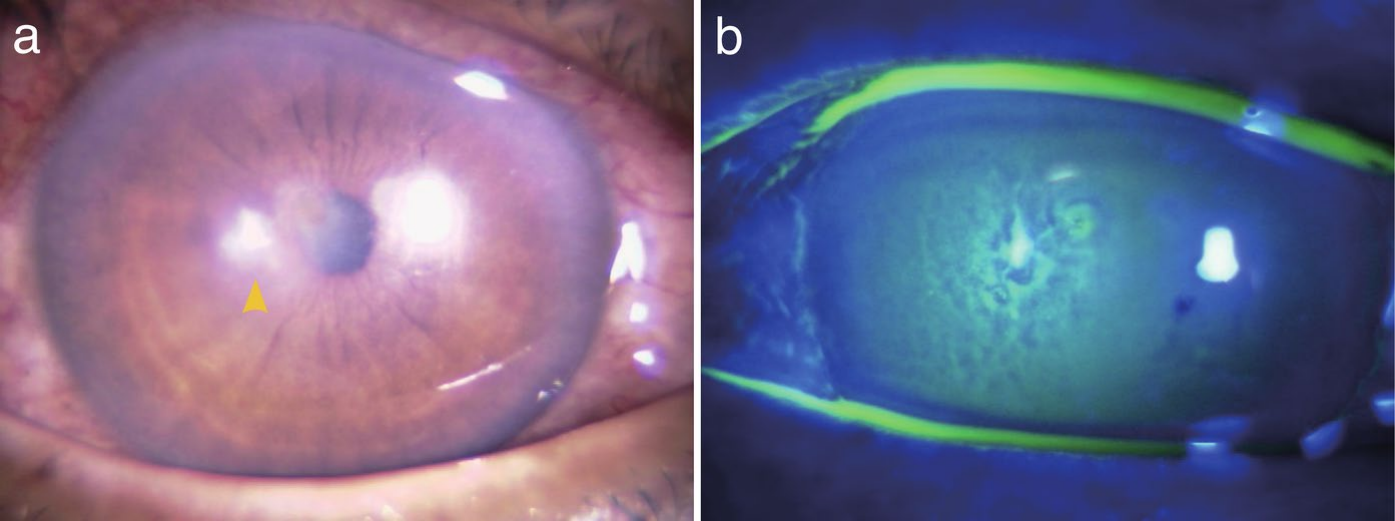
**a** Anterior segment photograph of Patient 2 at the initial examination, showing a 1-mm corneal infiltrate located in the paracentral area. **b** Fluorescein staining reveals an epithelial defect smaller than the area of corneal infiltration

A 63-year-old man sustained a right eye injury when foreign material entered his eye while cutting grass. He visited a local ophthalmologist 2 days after the injury and was prescribed levofloxacin and ofloxacin ophthalmic ointments. However, he developed ocular pain and decreased visual acuity with corneal infiltration, and he was referred to the Department of Ophthalmology at Hiroshima University Hospital 4 days after the initial injury. At presentation, the best-corrected visual acuity in the right eye was 20/50. An irregularly shaped infiltrate was observed in the paracentral cornea, accompanied by a small epithelial defect at the same location (Fig. 3a, b). Anterior segment optical coherence tomography revealed that the infiltrative lesion was confined to the superficial stroma without corneal thinning. Although direct microscopy and in vivo confocal microscopy did not reveal clear fungal elements, filamentous fungal infection was suspected based on the mechanism of injury and anterior segment findings. Topical natamycin was added to the treatment regimen, and the infiltrate had nearly resolved within 2 weeks of the initial visit, with complete epithelialization achieved. Topical betamethasone phosphate was then initiated, leading to healing with minimal scarring. Two months after the initial examination, best-corrected visual acuity in the right eye recovered to 20/16.

## Microbiological Findings

**Fig. 4:**
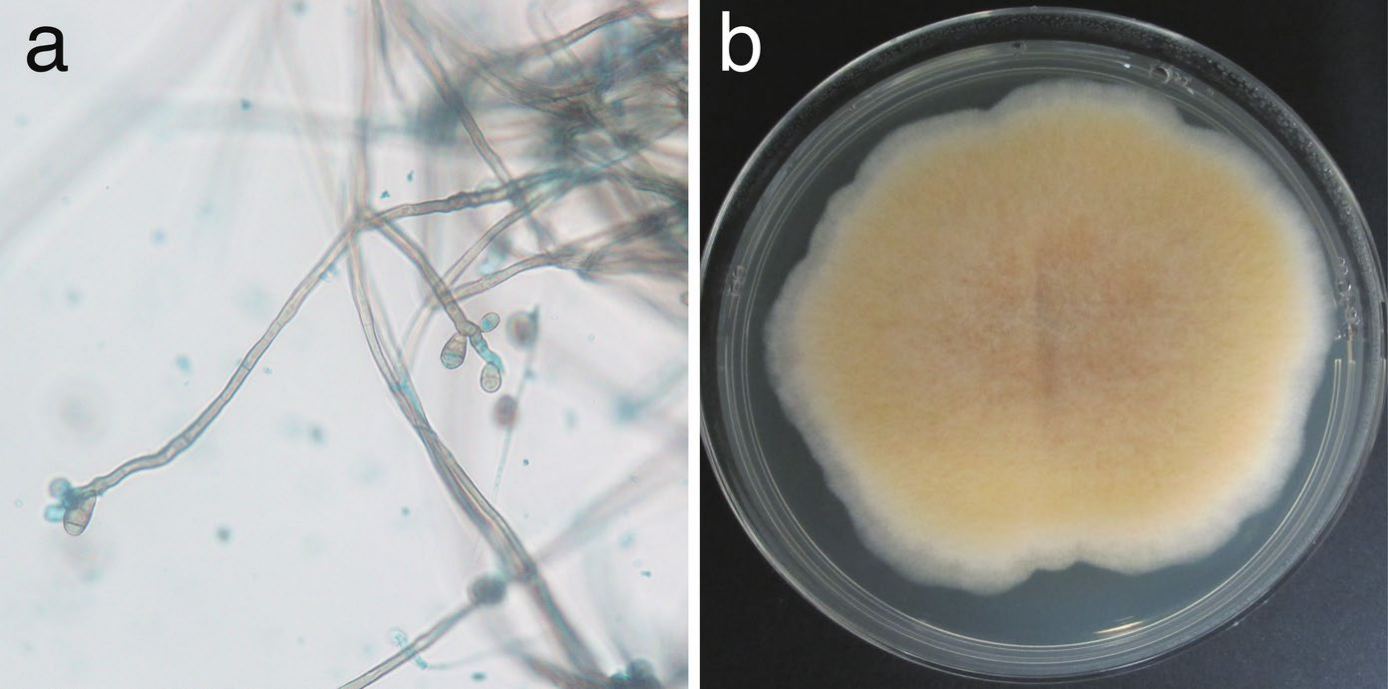
**a** Lactophenol cotton blue staining showing septate hyphae with curved, septate conidia. **b** Colony morphology on Sabouraud dextrose agar after 7 days of incubation at 25 °C, displaying a cream to pale yellow, velvety colony with a flat surface

The two isolates were morphologically and molecularly indistinguishable. The conidiogenous cells were terminal, brown, smooth, and bore darkened scars. The conidia were cylindrical to ellipsoidal, slightly curved, 3- to 5-distoseptate, pale brown to brown in color, and rounded at the apex (Fig. 4a). The cultured colonies had velvety surfaces (Fig. 4b). Based on these morphological features, the isolates were identified as belonging to the genus *Curvularia*. Sequence analysis of the translation elongation factor 1-alpha (*tef1-α*) gene showed 100% identity (929/929 bp) with *C. geniculata* strain NB871 (GenBank accession no. OP709948). The isolates were preserved as IFM 67522 and IFM 69711 at the Medical Mycology Research Center, Chiba University, through the National Bio-Resource Project, Japan (JPNBRP202228). Antifungal susceptibility testing was performed, and the data were interpreted as outlined in the Clinical and Laboratory Standards Institute M38-Ed3 Method [3]. Antifungal susceptibility testing was conducted for both isolates. In Patient 1’s isolate, amphotericin B, terbinafine, luliconazole, itraconazole, voriconazole, and ravuconazole all showed complete (100%) growth inhibition at concentrations of > 2 µg/mL. At 0.25 µg/mL, luliconazole, itraconazole, and ravuconazole showed 80% growth inhibition, while voriconazole achieved 80% inhibition at 0.5 µg/mL. Natamycin completely inhibited growth at 2 µg/mL. For the isolate from Patient 2, the minimum inhibitory concentrations were as follows: amphotericin B, complete inhibition at 0.25 µg/mL; natamycin, complete inhibition at 2 µg/mL. The 80% growth inhibition concentrations were as follows: terbinafine, 1 µg/mL; luliconazole, 0.06 µg/mL; itraconazole, 0.06 µg/mL; voriconazole, 0.5 µg/mL; and ravuconazole, 0.5 µg/mL. Both isolates were sensitive to natamycin at the same concentration (2 µg/mL), while Patient 2’s isolate demonstrated greater sensitivity to amphotericin B and azole antifungals than did Patient 1’s isolate.

## Discussion

This report describes two cases of keratitis caused by *C. geniculata*, a relatively uncommon cause of fungal keratitis, confirmed through molecular identification. Although *C. geniculata* keratitis has been reported in two cases since the 1960s [4, 5], our cases represent the first molecularly confirmed instances using genetic analysis. These findings underscore the clinical importance of accurate species identification within the genus *Curvularia* and offer valuable insights into the management of this rare condition.

The clinical presentation of *Curvularia* keratitis in our cases is consistent with previous reports in the literature. In a comprehensive 30-year study by Wilhelmus and Jones analyzing 43 cases of *Curvularia* keratitis, the clinical spectrum ranged from superficial feathery infiltrates of the central cornea to suppurative ulceration of the peripheral cornea [6]. The authors noted that *Curvularia* keratitis typically presents as a superficial feathery infiltration, rarely with visible pigmentation, which gradually becomes focally suppurative. Both of our patients exhibited characteristic feathery infiltrates—particularly prominent in Patient 1—further supporting these established clinical features.

The presence or absence of hypopyon is an important prognostic indicator in *Curvularia* keratitis. Hypopyon formation is relatively uncommon, occurring in only 12% of cases in the study by Wilhelmus and Jones [6], but when present, it was associated with a significantly increased risk of complications. Similarly, Khurana et al. reported hypopyon in 16.5% of 97 culture-proven *Curvularia* keratitis cases in India, with its absence linked to better visual outcomes [7]. Notably, neither of our patients developed hypopyon, and both achieved excellent final visual acuity (20/20 and 20/16, respectively), further supporting the established correlation between the absence of hypopyon and a favorable prognosis in *Curvularia* keratitis.

In our cases, molecular identification results became available approximately one month after initial presentation, including the time required for fungal culture (7–10 days), DNA extraction, PCR amplification, sequencing, and phylogenetic analysis. This timeframe, while typical for comprehensive molecular identification in our hospital setting, limits its utility for acute clinical decision-making. However, molecular confirmation remains valuable for definitive diagnosis, epidemiological surveillance, guiding therapy in refractory cases, and retrospective validation of empirical treatment decisions. Accurate species identification within the genus *Curvularia* has become increasingly important because of significant taxonomic revisions in recent years. Numerous cryptic species have been revealed through molecular phylogenetic studies [8–11], highlighting the insufficiency of morphological characteristics alone for reliable species determination. Many *Curvularia* species share overlapping morphological traits and conidial dimensions, making precise identification particularly challenging. In our cases, both isolates were definitively identified as *C. geniculata* sensu stricto through sequence analysis of the *tef1-α* gene, which showed 100% identity with the reference strain. Notably, initial analysis using only the internal transcribed spacer (ITS) region proved inadequate for definitive species-level identification. The ITS region, while useful as a universal fungal barcode, provides insufficient resolution for reliable species delimitation within the genus *Curvularia* due to low interspecific variation. Therefore, the translation elongation factor 1-alpha (tef1-α) gene was employed as it offers superior phylogenetic resolution and is now recognized as the primary barcode for *Curvularia* species identification [12]. The distinction between sensu lato and sensu stricto classifications is clinically relevant because different *Curvularia* species may exhibit varying pathogenicity, antifungal susceptibility profiles, and clinical outcomes.

The choice of antifungal therapy for *Curvularia* keratitis remains centered on natamycin, the only topical antifungal agent approved for ophthalmic use by the U.S. Food and Drug Administration [13, 14]. In a landmark study by Wilhelmus and Jones, all tested *Curvularia* isolates were inhibited by ≤ 4 μg/mL of natamycin, demonstrating excellent in vitro activity, and 78% of patients achieved a visual acuity of 20/40 or better [9]. Recent meta-analyses comparing topical natamycin with voriconazole have shown similar visual acuity outcomes at 2–3 months, although natamycin is associated with a lower risk of corneal perforation and therapeutic penetrating keratoplasty [15].

Our in vitro susceptibility testing showed that both isolates were sensitive to natamycin at 2 μg/mL, consistent with previous reports. However, there was notable variability in sensitivity to other antifungal agents between the two isolates, with Patient 2’s isolate demonstrating greater sensitivity to amphotericin B and azole antifungals than Patient 1’s isolate. This variability highlights the potential value of susceptibility testing in guiding therapy, particularly in cases that do not respond to initial treatment. In our approach, Patient 1 was treated with a combination of natamycin and voriconazole due to the larger, deeper infiltrate (2 mm, 50% stromal depth) with abundant fungal elements, while Patient 2 was successfully managed with natamycin alone for a smaller, superficial lesion. Both patients achieved complete healing and excellent visual outcomes, supporting the effectiveness of both strategies. Recent studies have reported synergistic effects between natamycin and voriconazole against *Curvularia* species in 23.1% of tested isolates [16], suggesting that combination therapy may be beneficial in selected cases.

Our findings suggest that molecular identification and susceptibility testing, while valuable, are not essential for successful management of *Curvularia* keratitis. Both cases responded excellently to empirical natamycin-based therapy initiated before species confirmation. In resource-limited settings, clinicians can achieve favorable outcomes using clinical judgment and basic microscopy to guide therapy, with treatment intensity (monotherapy versus combination) determined by clinical severity.

In conclusion, *C. geniculata* keratitis, though rare, can be effectively managed with appropriate antifungal therapy. Molecular identification is crucial for accurate diagnosis and species-level differentiation within the *Curvularia* genus. Both natamycin monotherapy and combination therapy with natamycin and voriconazole appear to be effective treatment options, with the choice potentially guided by clinical severity and antifungal susceptibility testing results.
